# Acceptability and adherence to Isoniazid preventive therapy in HIV-infected patients clinically screened for latent tuberculosis in Dar es Salaam, Tanzania

**DOI:** 10.1186/s12879-015-1085-7

**Published:** 2015-08-26

**Authors:** Grace A. Shayo, Candida Moshiro, Said Aboud, Muhammad Bakari, Ferdinand M. Mugusi

**Affiliations:** Department of Internal Medicine, Muhimbili University of Health and Allied Sciences, Dar es Salaam, Tanzania; Department of Epidemiology and Biostatistics, Muhimbili University of Health and Allied Sciences, Dar es Salaam, Tanzania; Department of Microbiology and Immunology, Muhimbili University of Health and Allied Sciences, Dar es Salaam, Tanzania

## Abstract

**Background:**

Proper adherence to isoniazid preventive therapy (IPT) may depend upon the results of tuberculosis (TB) screening test and patients’ understanding of their risk of developing active TB. We conducted a study to assess the acceptability, adherence and completion profile of IPT among HIV-infected patients who were clinically screened for latent TB Infection (LTBI).

**Methods:**

A multicenter observational study was conducted in Dar es Salaam, Tanzania between February 2012 and March 2014. HIV-infected patients 10 years or older were clinically screened using a validated symptom-based screening tool to rule out active TB. Patients found to have no symptoms in the screening tool were given 300 mg of isoniazid (INH) daily for 6 months. Patients were followed up monthly at the National and Municipal hospital HIV clinics for INH refill and assessment of treatment adherence. Adherence was defined as consumption of 90 % or more of the monthly prescription of INH.

**Results:**

All 1303 invited patients agreed to participate in the study. Of 1303 invited HIV-infected patients, 1283 (98.5 %) were recruited into the study. Twenty eight (2.2 %) did not complete treatment. Those who did not complete the treatment were exclusively adults aged 18 years or older, *p* = 0.302. The overall mean (±SD) adherence was 98.9 % (±2.9). Adherence level among children aged <18 years (92.2 %) was significantly lower than adherence level among patients aged 18–29 years (98.3 %), 30–49 years (98.8 %) and ≥ 50 years (98.5), *p*-value = 0.011. Sex, occupation, socio-economic status, duration of HIV infection, being on antiretroviral drugs (ARV) and duration of ARV use were not associated with adherence.

**Conclusion:**

IPT is highly accepted by HIV infected patients. Patients demonstrated high level of adherence to IPT. The level of adherence among children was slightly lower than that among adults. IPT non-completers were exclusively adults. Children might need adult supervision in taking IPT.

## Introduction

Tanzania is one of the 22 high burden countries (HBC) for tuberculosis (TB) according to the World Health Organization (WHO) [1] This TB epidemic is largely fueled by co-infection with human immunodeficiency virus (HIV). In 2011 the WHO recommended that patients infected by HIV in resource constrained settings should receive isoniazid preventive therapy (IPT) to reduce the incidence of TB [2]. IPT consists of isoniazid (INH) 300 mg. administered orally on a daily basis for 6 months. The recommendation is based on the evidence that IPT is both efficacious [3, 4] and relatively inexpensive [2, 5]. Despite the available evidence that IPT is efficacious many countries have failed to implement this recommendation. Concerns about IPT adherence, the cost of ruling out active TB, the safety of the long term use of INH and fear of the emergence of resistance to INH have all been cited as reasons for this failure [2]. Other countries have embraced it with Botswana having the largest number of HIV infected patients on IPT in the world [2].

Research on IPT provision has revealed variable rates of acceptance and completion of TB preventive therapy among populations at risk for TB. The overall completion rate for IPT was only 45 % in a 2010 study among patients attending chest clinics in New York City [6]. Alternatively, the IPT completion rate among HIV infected patients with a positive tuberculin skin test (TST) who received counseling and transport reimbursement was 87 % in a 2008 study in Dar es Salaam, Tanzania [7]. Adherence to IPT in TST-positive and non-TST-screened HIV infected subjects in Thailand in 2004 was 84.5 and 79.7 % by self- reporting, and 81.8 and 73.9 % by pill count monitoring [8]. In 2014 Berhe *et al.*, reported an IPT adherence rate of 86.5 % at one month among HIV infected patients in Ethiopia [9].

Good adherence to IPT has been linked to counseling before initiation of therapy, freedom to take INH publicly, and regular attendance at follow-up clinics [10]. Other relevant factors shown to affect IPT adherence in HIV infected patients include understanding of IPT rationale, beliefs about INH safety and concerns about potential INH side effects. Additionally, patient denial of HIV infection, HIV related stigma and concerns regarding concurrent use of highly active anti-retroviral therapy (HAART) are also relevant [11, 12]. Directly observed preventive therapy and a shorter duration regimen for preventive therapy have both been found to improve treatment completion rates [4]. Barriers to implementation of IPT have been found to be related to both patients’ factors as narrated above and health care system factors [13]. A 2012 study in Uganda reported a very low IPT completion rate at 33.6 % among HIV infected patients in a non-governmental facility. Factors reported to have been associated with non-completion included an age of less than 30 years and prior divorce, separation or widowhood [14].

We conducted the present study to assess acceptability, adherence and completion profile of IPT among HIV infected patients who were clinically screened for LTBI in Dar es Salaam, Tanzania.

## Methods

### Study design, site and population

This study was a prospective, observational multicenter study conducted at four clinics providing HIV-AIDS treatment services in Dar es Salaam. The study was conducted between February 2012 and March 2014. Patients being treated at these facilities were the study subjects. We conducted this present study to assess patient acceptance, adherence and completion profiles for IPT in HIV infected patients aged 10 years or older. Study participants included both those who had never treated with anti-retroviral (ARV) drugs and those either previously or currently being treated with ARV drugs.

All four HIV clinics are under the direction of the National AIDS Control Program (NACP) of Tanzania. All HIV-AIDS related treatment protocols and policies for Tanzania are directed and coordinated by the NACP. HIV treatment failures are managed at a higher-level referral clinic in Dar es Salaam that was not included in this study. Although the clinics in the study are sited at four different facilities within Dar es Salaam they function identically. All the clinics were urban set and were located at Muhimbili National Hospital (MNH) [a tertiary referral hospital which is also a teaching hospital for the Muhimbili University of Health and Allied Sciences], Amana and Temeke Municipal Hospitals which are secondary level hospitals that refer cases to MNH and the Pastoral Activities and Services for People with AIDS in Dar es Salaam Archdiocese (PASADA) Health Center. These HIV clinics operate five days a week and each sees approximately seventy to one hundred patients daily. All HIV infected patients are routinely seen monthly for clinical evaluation and receive refills of ARV drugs at the time of their visit.

The clinics at MNH, Amana, Temeke and PASADA were among the fourteen centers participating in a separate pilot study of IPT in HIV positive patients in Tanzania.

### Data collection and procedures

Eligible patients were HIV infected outpatients, ARV naïve or experienced, who were willing to stay in Dar es Salaam for at least 2 years and were aged 10 years or older. Trained nurses and doctors informed clinic patients about the present study that was to go hand in hand with the national IPT pilot study and invited them to participate in the study. Informed consent was obtained. Exclusion criteria were known alcohol abuse, a current or past history of hepatitis or other medical contraindications to INH therapy. Additional exclusion criteria were current or recent (within the past two years) TB treatment, active pregnancy, a history of treatment non-compliance or the presence of WHO clinical stage 4 AIDS [15].

Those who agreed to participate were screened to rule out active TB using the National Tuberculosis and Leprosy Program (NTLP) symptom-based screening tool [16]. The tool is comprised of 5 questions asking for the presence of cough for ≥ 2 weeks, fever for ≥2 weeks, hemoptysis of any duration, excessive night sweats for ≥ 2 weeks and noticeable weight loss or weight loss of ≥ 3 kg within 4 weeks. We previously demonstrated the tool had a sensitivity and specificity for identifying active TB of 71.4 and 75.9 % respectively in a HIV positive population being considered for IPT for LTBI. The positive and negative predictive values of the screening tool were 11.4 and 98.4 % respectively, with a false negative rate of 28.6 % [17].

Patients who presented with any of the symptoms in the screening tool were designated as being active TB suspects and underwent standard TB screening. Those confirmed with active TB were treated as per National TB guidelines [15]. Patients with no confirmed TB were eligible to be enrolled in the study. Patients who presented with none of the symptoms in the tool were considered to not have active TB and were assessed for IPT eligibility as above.

Eligible patients were consecutively enrolled into the study and initiated on IPT. The IPT comprised of INH 300 mg administered orally daily for 6 months in a non-supervised fashion. Patients were provided with a monthly dose of 30 tablets of INH to take home. Patients’ remaining pills were counted to ascertain adherence at each monthly follow up visit. A structured questionnaire was used to collect demographic, socioeconomic and clinical data at enrollment.

At each of the monthly visits the TB screening survey tool was repeated to identify those who could have possibly developed active TB during the follow up period. An interval clinical history was collected at each follow up visit to identify any new clinical developments or drug side effects. A thorough monthly examination of the respiratory system, lymphatic system, skin and mucous membranes, gastrointestinal tract (GIT), cardiovascular, central nervous system and musculoskeletal systems for any concurrent illness was conducted at each follow up visit.

Weight and body temperature were monitored in each visit. Weight was measured using an analogue scale (SECA) without shoes and was recorded to the nearest 0.5 kg. Temperature was measured in degrees Celsius using a digital thermometer.

### Definition of terms

Adherence was defined as consumption of 90 % or more of the monthly prescription of INH. IPT completion was defined as having received and consumed Isoniazid for a total of 6 months. Consent withdrawal meant a reported participant’s voluntary cessation of both IPT use and study participation. Lost to follow up was defined as absence from the clinic for at least two consecutive scheduled visits and efforts to track the patients were in vain.

### Ethical issues

Ethical clearance for the study was obtained from the MUHAS Institutional Review Board (Reference number MU/DRP/AEC/Vol.XVI/135). All the involved health facilities gave permission for the study to be conducted in the facilities. All patients consented to participate through a written informed consent. Patients aged < 18 years had their parents/guardians signed informed consent on their behalf having themselves provided verbal assent. Patients who screened positive to the screening tool were fully worked up to diagnose or rule out active TB. Upon diagnosis of active TB, patients were started on a full course of anti-TB treatment as per Tanzania guidelines [15]. All the data were handled with high confidentiality.

### Statistical analysis

Data analysis was performed using SPSS version 20. Proportions were used to describe the socio-demographics of patients. Adherence to IPT was calculated for each month using the pill count method. Tablets remaining in a particular month were subtracted from 30 (the tablets patients were given to carry home in each month). The difference of the two was then divided by 30 before being multiplied by 100 to obtain percentage adherence. The average of the adherence in the months the patients were on treatment was calculated for each patient. This is the parameter used in this study to describe adherence to IPT. Therefore if a patient stayed in the study for only 2 months, his adherence was the average adherence of the 2 months. Consumption of ≥ 90 % of monthly tablets was considered good adherence.

Socioeconomic status of respondents was assessed by the use of the factor analysis method [18]. A total of 16 variables were used to assess SES after being given weights. These variables included the following items: 1) family income per month; 2) type of the house walls (mud, bricks, blocks etc.); 3) roofing material used (grass, iron sheets, roof tiles etc.); 4) size of the house in terms of number of the people sleeping in a room; 5) floor type (mud, cement, wood, floor tiles etc.; 6) presence or absence of house ownership; 7) presence of electricity; 8) presence of tap water; 9) toilet type (pit latrine, Asian or European toilet etc.); 10) type of cooking fuel used (charcoal, fire wood, electricity, cooking gas, etc.); 11) car ownership; 12) television ownership; 13) refrigerator ownership; 14) bicycle or motorcycle ownership; 15) radio ownership; 16) fan ownership.

Non categorical variables had their means, frequencies and standard deviations calculated. Variables with low standard deviation were given a low weight as they had a minimal ability to differentiate SES of the patients. Variables with high standard deviation were given a high weight. The non-categorical variables were converted into binary variables and weighted. A Principal Component Analysis (PCA) was then used to derive factor scores for every weighted variable using data reduction and regression method [18]. Variables that had a positive factor score were associated with a high socioeconomic status while those with a negative factor score were associated with a low socioeconomic status. These factor scores were analyzed to generate the 3 categories of socioeconomic status. Cross tabulations and Pearson’s Chi-square test were used to obtain the associations and strength of relationship between the independent and the dependent variables. Chi square test was used to compare adherence to IPT across different socio-demographic and clinical characteristics. P value of < 0.05 was considered significant.

## Results

**Fig. 1:**
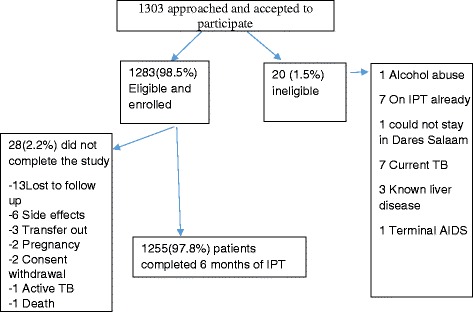
Patient recruitment and retention flow chart

A total of 1303 patients were consecutively invited to participate in the study. All 1303 (100 %) patients accepted to participate. Of the 1303 patients, 20 patients were ineligible for the study due to alcohol abuse in 1 patient, 1 patient could not stay in the city for the study duration, 7 patients were already on IPT, 7 patients were either on TB treatment or had finished TB treatment within 2 years, 3 patients had known liver disease and 1 patient had stage 4 HIV disease. A total of 1283 (98.5 %) were eligible for the study and were offered IPT. At the end of 6 months of IPT 1255 patients had successfully completed 6 months of treatment. Non-completers numbered 28. Reasons for non-completion of IPT were as follows: 2 withdrawals of consent (these withdrew from the study and stopped using IPT), 13 lost to follow up, 1 death, 6 side effects, 3 transfer out, 2 pregnancy and 1 active TB (Fig. 1). All the 6 cases who did not complete 6 months of IPT due to side effects stopped using IPT on their own against physicians’ advice. The side effects were mild rash in 4 patients, burning sensation of limbs in 1 patient and numbness of the feet in 1 patient.

Females were the majority, constituting 77.5 % (994/1283) of the patients. The majority of the patients (71.6 %) were aged 30–49 years old. There were 51 (4.0 %) minors (aged less than 18 years). The mean (± SD) age of all patients was 39.37 (±10.51). Self-employed patients were the majority (745/1283, 58.1 %) as were patients who attained only primary level education (289/1283, 69.5 %). The vast majority of patients were of low to medium socio-economic status (1218/1283, 94.9 %). More than half (55.2 %) of patients had lived with HIV infection for 13–60 months since diagnosis (598/1283). A total of 655/1283 (52.9 %) had current CD4+ cell counts > 350 cells/μl. More than two thirds of the patients (76.5 %) were on antiretroviral treatment (982/1283). A total of 494/920 (53.7 %) of those on antiretroviral treatment had been on ARV therapy for 13–60 months. See Table 1.

**Table 1:** Characteristics of the study participants, *N* = 1283

Characteristic	Number	Percentage
Sex: Females	994	77.5
Males	289	22.5
Age groups (in years)		
< 18	51	4.0
18-29	120	9.4
30-49	918	71.6
50+	194	15.1
Education (^a^ *N* = 416)		
No formal education	22	5.2.
Primary education	289	69.5
Secondary/post-secondary education	104	25.3
Occupation		
Unemployed	398	31.0
Employed	140	10.9
Self employed	745	58.1
Socio-economic status		
Low	520	40.5
Medium	698	54.4
High	65	5.1
HIV duration in months (^b^ *N* = 1084)		
0-12	241	22.2
13-60	598	55.2
> 60	245	22.6
ARV use		
Yes	982	76.5
No	301	23.5
Duration of ARV use in months (^c^ *N* = 920)		
0-12	229	24.9
13-60	494	53.7
> 60	197	21.4
Current CD4 counts in cells/ μl (^d^ *N* = 1239)		
0-200	245	19.8
201-350	339	27.4
> 350	655	52.9

^a^Level of education was available for only 416 patients

^b^Only 1084 patients had known duration of HIV infection since diagnosis

^c^Only 920 of 982 patients on ARVs could recall the date they commenced ARVs

^d^Only 1239 patients had their current CD4 available

One patient withdrew consent after receiving the 1^st^ monthly dose of INH and had not used the drugs as was noted in the 2^nd^ month of follow up. This patient was excluded from the analysis of IPT adherence leaving 1282 patients in this cohort. The overall mean (±SD) for IPT adherence was 98.9 (±2.9), a strikingly high percentage. The IPT adherence level among children aged <18 years (92.2 %) was significantly lower than adherence level among patients aged 18–29 years (98.3), 30–49 years (98.8) and those 50 years or older (98.5 %) with a p-value = 0.011. Sex, occupation, level of education, socio-economic status, HIV duration since diagnosis, ARV use status, duration of ARV use and current CD4 counts were not associated with adherence. See Table 2.

**Table 2:** Factors associated with adherence to IPT, N^a^ = 1282

Characteristic	Total	Adherent patients	*P*- value
		Number (%)	
Sex: Males	289	284 (98.3 %)	
Females	993	975 (98.2)	0.926
Age in years			
<18	51	47 (92.2)	
18-29	120	118 (98.3)	
30-49	917	903 (98.5)	
≥50	194	191 (98.5)	0.011
Education (^a^ *N* = 415)			
No formal education	22	22 (100)	
Primary education	289	285 (98.6)	
Secondary/post-secondary education	104	102 (98.1)	0.780
Occupation			
Unemployed	398	388 (97.5)	
Employed	140	136 (97.1)	
Self employed	744	735 (98.8)	0.173
Socio-economic status			
Low	520	509 (97.7)	
Medium	697	688 (98.7)	
High	65	63 (96.9)	0.489
HIV duration since diagnosis (months) (^b^ *N* = 1084)			
0-12	241	235 (97.9)	
13-60	598	588 (98.3)	
>60	245	239 (97.6)	0.746
ARV use			
Yes	981	964 (98.3)	
No	301	295 (98.0)	0.766
ARV duration in months (^a^ *N* = 919)			
0-12	229	224 (97.8)	
13-60	494	489 (99.0)	
>60	196	191 (97.4)	0.266
Current CD4 counts in cells/ μl (^a^ *N* = 1238)			
0-200	245	241 (98.4)	
201-350	339	334 (98.5)	
>350	654	642 (98.5)	0.914

^a^One patient was not analyzed for adherence

^b^Only 1084 patients had known duration of HIV infection since diagnosis

**p-value for trend

Table 3 presents socio-demographic and clinical characteristics of 6 month IPT non-completers. Non-completers were exclusively adults aged 18 years or older; however this was not statistically significant. Adults younger than 30 years were more likely to be non-completers than other adult age groups but this was not statistically significant.

**Table 3:** Socio-demographic and clinical characteristics of non-completers

Characteristic	Total	Non- completers	*P*-value
		Number (%)	
Sex: Females	994	24 (2.4)	
Males	289	4 (1.4)	0.291
Age groups (in years)			
< 18	51	0 (0)	
18-29	120	6 (5.0)	
30-49	917	19 (2.1)	
≥ 50	194	3 (1.5)	0.111
Occupation			
Unemployed	398	9 (2.3)	
Employed	140	3 (2.1)	
Self employed	745	16 (2.1)	0.992
Education (*N* = 416)			
No formal education	22	0 (0)	
Primary education	289	8 (2.8)	
Secondary/post-secondary education	105	5 (4.8)	0.415
Socio-economic status			
Low	521	15 (2.9)	
Medium	697	10 (1.4)	
High	65	3 (4.6)	0.09
HIV duration in months (*N* ^a^ = 1084)			
0-12	241	7 (2.9)	
13-60	598	9 (1.5)	
> 60	245	7 (2.9)	0.295
ARV use			
Yes	982	20 (2.0)	
No	301	8 (2.7)	0.519
Duration of ARV use in months (*N* ^b^ = 920)			
0-12	229	5 (2.2)	
13-60	494	9 (1.8)	
> 60	197	5 (2.5)	0.828
Current CD4 counts in cells/μl (*N* ^c^ = 1238)			
0-200	245	7 (2.9)	
201-350	339	6 (1.8)	
> 350	655	15 (2.3)	0.682

^a^Only 1084 patients had known duration of HIV infection since diagnosis

^b^Only 920 of 982 patients on ARVs could recall the date they commenced ARVs

^c^One patient was not analyzed for adherence

## Discussion

The level of acceptance and completion rate of IPT has been found to be very high in the present study. The level of acceptance and completion were 99 and 87 % respectively in another study done in the city of Dar es Salaam by Munseri et al., 2008 [7]. The high acceptability of IPT in the two studies indicates that HIV infected patients are ready to accept interventions that are said to improve their health. A study in Rio de Janeiro, Brazil found significantly higher completion rate among patients already on ARV than those not on ARV [19]. A prior study in Ethiopia found that individuals who received pre-treatment counseling from health care workers were 8 times more likely to be adherent to IPT [10]. The participants of the present study did not receive any counselling before initiation of IPT.

In the present study the non-completion rate was very low at 2.2 %. This rate is comparable to the rate in the Thailand study that found non-TST screened patients had a dropout rate at 1.7 % compared to 6.3 % in TST screened subjects (8). However, this rate is substantially lower than the non-completion rate of 13 % noted by Munseri *et al.*, in Tanzania [7]. ARV use in Tanzania 10 years ago was substantially less than during the current study period due to the increased availability of medications.

The low non-completion rate we found can partly be explained by the high percentage of anti-retroviral drug treatment in our study group. Patients had an incentive to come to the clinic monthly already that was independent of IPT therapy thus removing any additional travel and time burden related to acquiring monthly IPT medication. A retrospective study in Uganda found an IPT non-completion rate of 58.1 % among HIV infected patients seen every other month in 2 non-governmental organization voluntary counselling and testing clinics who provided IPT in the form of 300 mg of INH daily for 9 months [14].

In the present study, non-completers were exclusively adults aged 18 years or older. The 18–29 year old age group had the highest rate for non-completion. Special interventions may need to be devised to retain them in IPT treatment. Patients with a high socioeconomic status showed a higher percentage of non-completers than other SES categories, however this was not statistically significant. Mindachew M *et al.*, 2011 could not find the association between monthly income and IPT adherence in Ethiopia [10]. The percentages of non-completers did not differ across other clinical characteristics. Adults of 18 years or older were also found to be the majority of non-completers in the study in the New York City among patients attending outpatient chest clinics [6]. This finding remains unexplained.

Generally, adherence to treatment was very good. Good adherence might have been a result of patients acting in the accordance to the directives to take INH daily. The high adherence level promises good outcomes once IPT provision becomes a country-wide recommendation in Tanzania. However, in the present study participants with a known history of treatment non-compliance were excluded, possibly creating a selection bias which favored high adherence.

Adults were significantly more adherent to IPT than were children. In Tanzania, children 10 years or older are usually informed about their HIV status and trained to be independent of their guardians when it comes to HIV care and treatment. A number of children less than 18 years are already taking their ARVs independently. A study in Botswana found that compared to controls, non-adherent cases were more likely to be young [20]. Although children were less adherent than adults in the present study, all the children completed the 6 months of treatment. This may suggest that children are likely to use TB preventive therapy correctly when well supervised.

## Conclusion

IPT is highly accepted by HIV infected patients. Patients demonstrated a high level of adherence to IPT therapy. The level of adherence among children was slightly lower than that among adults. The high acceptability and adherence levels make a compelling case that HIV infected patients are ready to accept and adhere to IPT. Adults, especially those aged less than 30 years were more likely to be IPT non-completers than were children. Children’s adherence to IPT was acceptably high, however children might need adult supervision on taking IPT to obtain as high an adherence rate as the adults.

## Study limitation

ARV naïve patients were requested to come to the clinic on a monthly basis for clinical evaluation and INH refill despite the fact that some of them had their normal clinical attendance 2 monthly. This could have contributed to indifference in adherence level between ARV naïve and ARV-experienced patients.
